# Investigation of Pre- and Postnatal Abnormalities Caused by Prenatal CMV Infection—Systematic Review

**DOI:** 10.3390/children12050607

**Published:** 2025-05-06

**Authors:** Virág Bartek, Artur Beke

**Affiliations:** Department of Obstetrics and Gynecology, Semmelweis University, 1085 Budapest, Hungary; bartek.virag@phd.semmelweis.hu

**Keywords:** CMV, prenatal, infection, ultrasound

## Abstract

Background/Objectives: CMV (cytomegalovirus) is associated with several developmental disorders. The incidence of congenital cytomegalovirus infection is around 1%, depending on the region. Previous prospective studies have shown that certain ultrasound findings are predictive factors for prenatal CMV infection. Methods: During this systematic review, we searched PubMed and Embas. Out of 569 results, 19 met our search criteria (we included cases where prenatally positive amniocentesis PCR for CMV was performed or autopsy confirmed the CMV diagnosis). A total of 237 cases were reported from 19 studies. Results: In 64 cases, abortion or perinatal death occurred. The most common prenatal abnormalities were small for gestational age (n = 47), ventriculomegaly (n = 51), and hyperechogenic bowels (n = 39). A subependymal cyst was the most common prenatal MRI abnormality (n = 20). Hearing loss was observed in 61 cases (42 mild, 19 severe). Among prenatal signs, we found a correlation between hearing loss and ventriculomegaly (Fisher’s exact test, *p* = 0.0052). The most common neurological complication was speech delay. We were able to demonstrate a prenatal association with neurological complications and subependymal cyst (Fisher’s exact test, *p* = 0.00003547), but this pattern could only be reliably seen with MRI. Conclusions: In prenatally diagnosed CMV infection, ultrasound signals may be suitable for estimating the outcome. Conducting a prospective study and establishing a score would be worthwhile for its clinical application. In cases of ultrasound abnormalities and suspicion of CMV, it is worth performing a prenatal MRI, even in everyday practice.

## 1. Introduction

Cytomegalovirus (CMV) is a member of the herpesvirus family. It primarily attacks endothelial cells and is a neurotropic virus. It has a potentially lifelong incubation period; therefore, an active infection may arise from a primary infection or from a non-primary infection caused by reinfection and/or reactivation. It is asymptomatic in immunocompetent individuals, while in immunocompromised individuals and pregnant women it can cause serious infection. Also, it is associated with the development of numerous developmental disorders [1]. Congenital CMV infection occurs via vertical transmission across the placenta, with a transmission rate of 30–40% for primary infection and around 1% for non-primary maternal infection [2].

Congenital cytomegalovirus infection occurs in 0.4–0.6% of live births in developed countries, and over 1% in developing countries [3]. The risk of perinatal death is increased in both symptomatic and asymptomatic cases, although different studies report different levels of risk (0.4–0.8%) [4].

As cytomegalovirus primarily attacks endothelial cells [5,6], the organ systems affected are diverse.

The most characteristic ultrasound signals were summarized by Benoist et al. and Malinger et al. [7,8]. The most characteristic intracranial abnormalities are ventriculomegaly, increased periventricular echogenicity or halo, periventricular pseudocysts, fetal intracranial calcification (especially periventricular calcification), heterogeneous brain parenchyma, microcephaly, and intraventricular adhesions. Other “minor” abnormalities may also occur, such as polar temporal lesions (dilation of temporal horns, WM T2-weighted signal hyperintensity, and cystic lesions), subependymal cysts, and intraventricular septa [9]. Extracerebral abnormalities are generally nonspecific, but with appropriate history and clinical presentation, they may indicate infection. These include IUGR/SGR (intrauterine growth restriction/retardation, small for gestational age), hepatosplenomegaly, hyperechogenic intestines, pericardial or pleural fluid, ascites or hydrops, and cardiomegaly.

With the spread of prenatal MRI, increasingly accurate examinations have become possible, so prenatal brain MRI suggests CMV infection by white matter lesions, late myelination, periventricular and temporal horn cysts, and migration disorders (lissencephaly, polymicrogyria, etc.) [10]. MRI can be the gold standard in postnatal diagnosis, but it is also increasingly used in prenatal diagnosis. MRI is better at detecting temporal polar lesions and polymicrogyria. However, the clinical significance of these minor signs is questionable, as there is no reliable study of the relationship between minor lesions and long-term follow-up [9,11]. With the development of radiology, prenatal MRI is now as informative as neonatal MRI [10].

Neurotropic viruses primarily affect the central nervous system, resulting in varying degrees of mental retardation, epilepsy, and cerebral palsy, and varying degrees and sides of hearing impairment or loss (uni- or bilateral) [12].

The timing of infection and seroconversion has a major impact on the course of infection and the development of malformations. The current position is that infection in the first trimester carries the highest risk. The results are more uncertain regarding later infection [11,13,14,15].

After the neonatal period, prenatal CMV detection by serological and virological methods is very difficult, and an alternative is retrospective examination of CMV DNA on dried blood spots collected in the first few days of life [16]. In postnatally symptomatic cases, neonatal cranial MRI is recommended to assess the outcome. A correlation between intracranial calcification and white matter abnormalities and lower intelligence has been demonstrated, but there are also case reports to the contrary [17,18,19].

Although the association between prenatal imaging findings and CMV is well-known and documented, there is relatively little literature on prognosis and its association with prenatal testing. Our aim in this study was to compile and review studies that specifically address prenatal imaging findings and postnatal outcome.

## 2. Materials and Methods

This study was a systematic review and was conducted following PRISMA 2020 guidelines [20].

During the systematic review, we searched PubMed, Web of Science, and Embase. We examined all available scientific articles, case studies, and retrospective and prospective cohort studies published after 1995. Those that matched the search terms “cytomegalovirus”, “ultrasound and/or MRI”, “prenatal”, “congenital”, and/or “infant” were reviewed. After a detailed analysis of the abstracts, we excluded those that were not written in English or whose English transcripts were not available, summary studies, meta-analyses, experimental descriptions, or descriptions of animal models.

**Figure 1:**
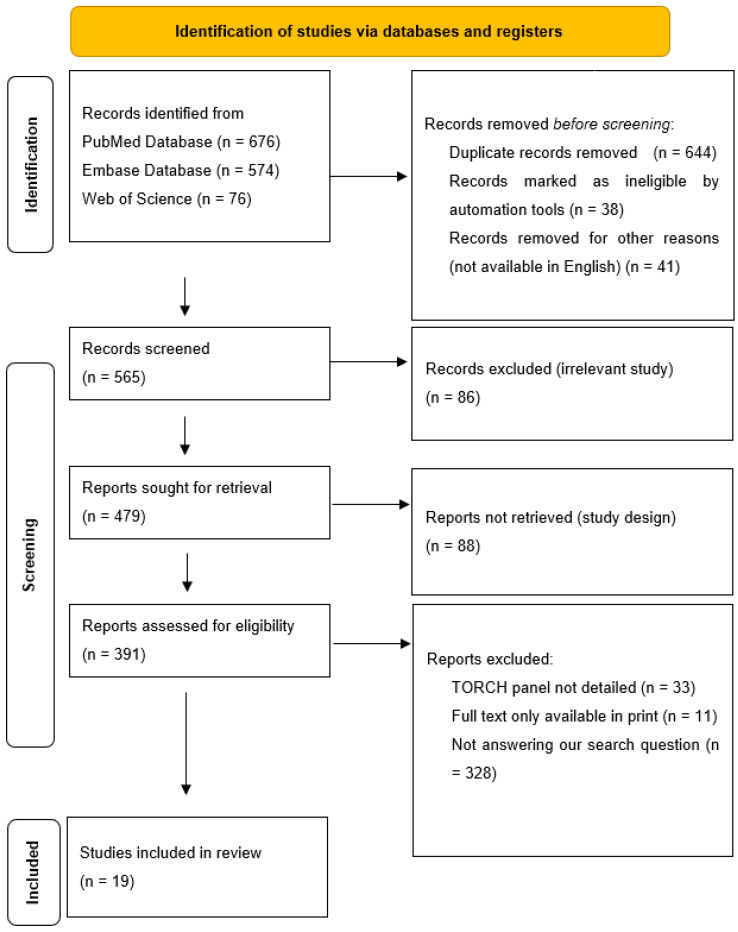
Elimination process.

While processing the abstracts, we excluded all articles that did not adequately answer our research question, i.e., what prenatal ultrasound abnormalities are associated with congenital cytomegalovirus, and what postnatal abnormalities are associated with these? We excluded all articles from the processing where cytomegalovirus and other TORCH (“toxoplasmosis, rubella cytomegalovirus, herpes simplex, and HSV”) infections were described together. The decision process is described in Figure 1.

The last day of the search was the 20 December 2024. The search and the elimination process was carried out by both authors. The data collection was conducted by Virag Bartek. The elimination process was performed using Rayyan (https://www.rayyan.ai/, last accessed on 5 January 2025).

The data were collected and analyzed in Excel (v. 16.89.1 (24091630)). The collected data were as follows: number of patients, author, publication year and place, ultrasound abnormalities prenatally (hydrops, hyperechogenic bowels, intracranial calcification, microcephaly, ventriculomegaly, hepatosplenomegaly, placentomegaly, oligohydramnios, IUGR/SGR, other), MRI abnormalities (pseudocyst, periventricular cyst, subependymal cyst, hypoplastic cerebellum, other), TOP (termination of pregnancy)/perinatal exitus, postnatal complication—deafness, neurological postnatal complication, postnatal imaging results, and long-term follow up.

**Figure 2:**
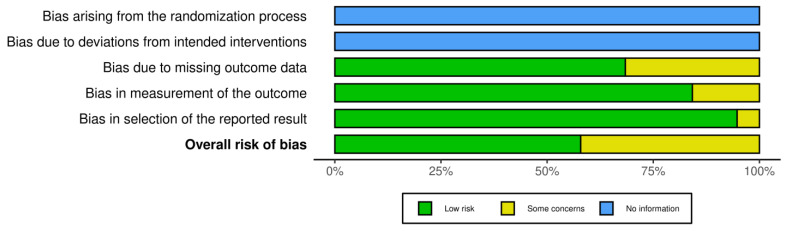
Risk bias for the statistical analysis due to the low number of data we used for Fisher’s exact test.

Risk bias was carried out via robvis (RobVis 2008, McGuinness, LA, Higgins, JPT. Risk-of-bias VISualization (robvis)), accessed on 14 April 2025. There was no bias arising from the randomization or the deviations of intended interventions, as there was not any randomization in our systematic review. Also, we selected studies with the same design (mostly individual case reports). The risk bias was performed by both authors. The risk was considered low if there was a detailed prenatal examination report with ultrasound and MRI report, and when the follow-up was available. Also, if there was a case of perinatal exitus, the autopsy report was available. When the ultrasound report or the MRI report was not fully available, or the follow-up was available only for the perinatal period, and in cases where the autopsy report was not fully available, and the authors only described the findings, the study was categorized as “some concerns”. Cases where the ultrasound or MRI report was completely missing, and neither a follow up nor an autopsy had been conducted, were excluded from the study. The risk bias is described in Figure 2.

## 3. Results

A total of 237 cases from 19 studies were included. Cases were included where prenatally positive amniocentesis PCR (polymerase chain reaction) for CMV was performed, or autopsy confirmed the CMV diagnosis.

Of the 237 cases, 64 resulted in termination of pregnancy or perinatal death.

The most common ultrasound abnormalities were tabulated and their association with outcome was examined (Table 1). The ultrasound abnormalities are summarized in Table 2.

**Figure 3:**
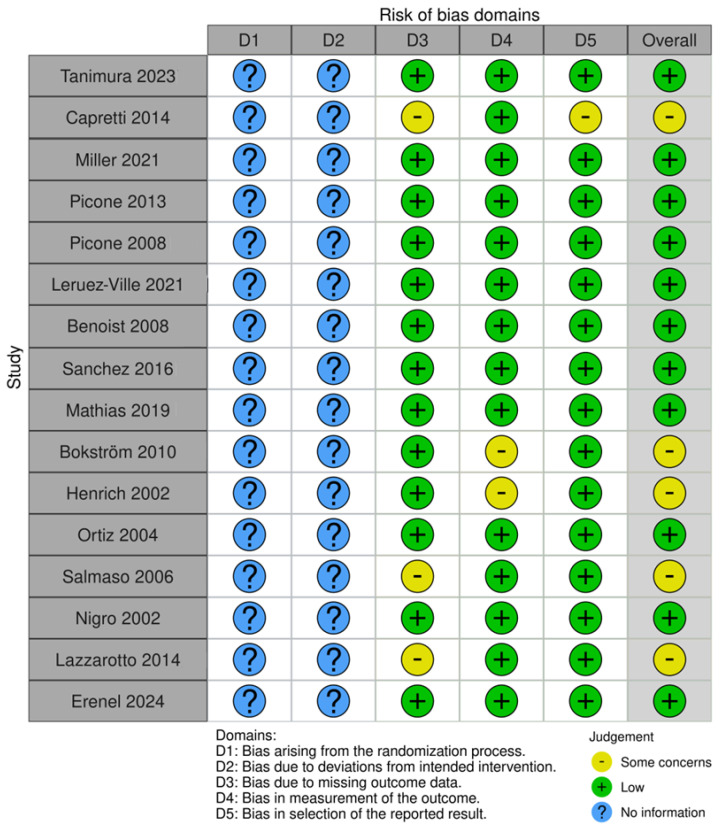
Risk bias of all studies with ultrasound anomalies.

The risk bias of all included studies is presented in Figure 3.

Among other abnormalities, cardiomegaly was described in two cases, hyperechogenic kidneys in two cases, and corpus callosum abnormalities in one case.

Among the MRI abnormalities, we found the following (Table 3):

We examined ENT (ear, nose, and throat) complications. We considered these to be mild if the hearing loss was unilateral or bilateral, and mild, and severe if the hearing loss was bilateral or unilateral, and severe or accompanied by complete deafness (Table 4). All data are given on 173 cases, as 64 cases ended in TOP or perinatal death.

We used Fisher’s exact test to examine whether there was a correlation between prenatal ultrasound abnormalities and deafness. We examined the five most common prenatal abnormalities. Since we did not obtain significant results even in the case of hydrops, we did not examine the others due to the low number of cases. We obtained a significant result for ventriculomegaly, meaning that there was a correlation between prenatally diagnosed ventriculomegaly and deafness (Table 5).

We examined whether there was a correlation between the severity of ventriculomegaly and the severity of deafness (Fisher’s exact test), but this could not be confirmed (0.11076711). The severity was based on the following: mild, the ventricles were between 10 mm and 12 mm; moderate, the ventricles were between 13 mm and 15 mm; and severe, the ventricles were more than 15 mm wide. As we did not get a significant result, we did not carry out any more tests or investigations related to severity.

We investigated whether there was a correlation between the prenatally indicated abnormality and later neurological complications (Table 6). We found a small number of cases with postnatal neurological complications and examined these together.

Due to the small number of cases, we examined the relationship between ventriculomegaly and subependymal cyst, where we obtained a significant result with Fisher’s exact test for the subependymal cyst (Table 7). However, due to the small number of cases, the clinical relevance of this is questionable.

## 4. Discussion

Cytomegalovirus infection is a worldwide problem in prenatal screening and care. With the spread of imaging methods, adequate prenatal screening and treatment can be performed, thus preventing the development of later complications.

As can be seen from the literature review, although prenatal cytomegalovirus infection imposes a great burden on both healthcare and pregnant mothers, few of the studies dealing with it are clinically relevant. This is because prospective studies are difficult to design and blinded studies are ethically unfeasible. Therefore, our current knowledge is mainly limited to retrospective and descriptive studies. Our review aimed to summarize the available scientific papers from the past twenty years, specifically focusing on long-term outcomes. Since prenatal imaging signs of cytomegalovirus have been well documented, we aimed to determine what findings and relationships could be discovered regarding prognosis.

Tanimura et al. in 2017 prospectively determined that specific ultrasound signs were independently predictive factors (odds ratio [OR], 31.9; 95% confidence interval [CI], 8.5–120.3; *p* < 0.001) of congenital cytomegalovirus infection in IgM-positive pregnant women [35]. Because ultrasound is highly operator- and device-dependent, it is difficult to determine the exact specificity and sensitivity. Previous studies have shown that diagnostic sensitivity is much lower for screening ultrasounds than for targeted testing after a confirmed infection [20,36]. This may be because CMV infection has nonspecific ultrasound signs, and in cases of confirmed infection, it is easier for the sonographer to look for common ultrasound abnormalities [24].

Among the specific (intracranial) and nonspecific (extracranial) ultrasound signs, the most common in our study were ventriculomegaly (n = 51), IUGR/SGR (n = 47), and hyperechogenic intestines (n = 39), which is consistent with the literature data [7,8].

The most common complications were hearing loss and neurological complications. In the case of hearing loss, the degree of hearing loss was mild in 42 cases, while in 19 cases the degree of hearing loss was severe. Among the neurological complications, epilepsy developed in 4 cases, cerebral palsy in 6 cases, motor delay in 8 cases, cognitive delay in 8 cases, and verbal delay in 15 cases. ADHD (attention-deficit/hyperactivity disorder) was described in four cases.

Prenatal CMV infection is the leading enviromental cause of sensorineural hearing loss in the United States. Although the association between prenatal CMV infection and postnatal hearing loss has been known for more than fifty years, it is still not possible to assess prenatally whether, and to what extent, the affected child will be affected after birth [37]. Craeghs et al. investigated whether there was a link between postnatal neurological abnormalities confirmed by neonatal imaging, and postnatal hearing loss. They included 411 patients, 40% of whom were symptomatic at birth. They found a significant association between newborns with ultrasound and/or MRI abnormalities and symptomatic hearing loss, but not between late-onset hearing loss and imaging abnormalities [38]. Corazzi and colleagues found a correlation between postnatal brain MRI abnormalities and sensorineural hearing loss [39]. There is little literature available on the relationship between prenatal imaging abnormalities and hearing loss. Lipitz et al. found no significant association between prenatal imaging abnormalities and hearing loss (*p* = 0.084, 0.109, and 0.176, respectively) [40].

Attention-deficit/hyperactivity disorder (ADHD) is a heterogeneous disorder with both predetermined, genetic and environmental causes. Its exact pathomechanism is unknown. It has shown an increasing trend in recent years, and is therefore—understandably—an area of active research [41]. In their meta-analysis, Chun-yan Zhu et al. found that prenatal infection slightly increased the risk of ADHD (OR, 1.25; 95% CI, 1.09, 1.44; *p* < 0.0001; I^2^ = 92.9%, *p* < 0.0001), but the results were not significant in studies examining siblings, so prenatal infection can only partially explain the increased risk. Chun-yan Zhu et al. did not specifically examine cytomegalovirus [42]. Due to the small number of cases (four cases), we were unable to significantly examine the relationship between ADHD and prenatal imaging studies. Nis Borbye-Lorenzen and colleagues found that high anti-CMV levels increased the risk of later ADHD development [43].

The relationship between prenatal ultrasound signals and postnatal outcome is currently unclear. In our study, we found a significant association between ventriculomegaly and deafness, but the sample size was small, so we could only perform statistical testing with Fisher’s exact test, which did not show a strong statistical association. Letouzey et al., in their 2017 retrospective study, found no association between neurological outcome and the degree of isolated ventriculomegaly (n = 21) [44]. In their 2013 cohort study (n = 23) Alacron et al. examined several prenatal factors and found that combining prenatal imaging studies, CSF (cerebrospinal fluid) β2-m levels, and head circumference could identify a prognostic factor for neurological outcome [45].

As can be seen from the above, doing prenatal studies is very difficult, both from an ethical and from a research design perspective. As can be seen from the above descriptions, the case numbers were generally very low, and there was great heterogeneity in the studies described regarding which prenatal factors were analyzed (imaging studies, antibodies, prenatal treatment) and what type and length of follow-up was performed. Interruption of follow-up is also common since, to achieve significant results, follow-up should be carried out at least until school age. Nevertheless, this field is worth addressing. In particular, due to the heterogeneity of the sample, and the examination of several different factors, it would be worthwhile to develop a scoring system based on a large, multicenter, prospective study, if possible, but at least on a retrospective study with long follow-up, which summarizes all the factors described above. Based on these factors, an estimate of the expected prognosis could be given in the prenatal period. The current literature on this topic also supports the recommendation for a multimodal, multifactorial prognostic factor [23,46]. This is not only important for the pregnant woman to make adequate decisions but also provides guidance for the team providing postnatal care as to what professionals need to be involved and what improvements can be introduced. It would also give a more accurate screening method.

## 5. Conclusions

In the case of prenatally diagnosed CMV infection, the prognosis can be estimated based on ultrasound signs. Since it is a phenotypically diverse disease, it would be worthwhile to develop a score system for risk estimation and appropriate clinical application by examining several different factors, possibly with a prospective study.

Certain ultrasound signs, especially in combination, suggest the diagnosis of prenatal TORCH infection, especially CMV, in which case it may be worth recommending amniocentesis to the pregnant woman even in the case of doubtful serological findings. If the ultrasound abnormalities are unclear, prenatal MRI can also be recommended as an additional diagnosis, as it provides a much clearer picture of the extent of the disease.

There is probably a correlation between abnormalities affecting the nervous system (ventriculomegaly and subependymal cyst) and prognosis, which we could confirm, but only with weak statistical tests due to the small number of cases. We plan to investigate this further with a larger number of cases.

Proper prenatal diagnosis not only contributes to adequate prenatal treatment, but also it also contributes to the pregnant woman being able to make the right decision regarding the outcome of her pregnancy. If delivery occurs, proper diagnosis and risk assessment contribute to the newborn being treated by an interdisciplinary team experienced in CMV infection. Early treatment is essential for a positive outcome.

## Figures and Tables

**Table 1:** Summary of data obtained during the literature review. For ease of interpretation, cases where there was only MRI abnormality but no ultrasound abnormality are not presented separately.

			US Abnormalities										MRI Abnormalities			
Author		Year	Hydrops	HB	Calcification	Microcephaly	Ventriculomegaly	Hepatosplenomegaly	Placentomegaly	Oligohydramnios	IUGR/SGR	Other	Pseudocyst	PV Cyst	SE Cyst	Hypoplastric Cerebellum
Tanimura et al. [21]		2023	*								*	CM, PH				
										*	*					
											*					
			*			*	*				*		*			*
				*			*	*			*	CM				
							*				*		*			
					*		*	*			*					*
							*				*					*
							*						*			
			*				*	*	*							
			*	*			*	*					*			
							*	*								
							*									
			*		*		*	*	*							*
			*				*	*								
			*	*			*	*	*							
			*	*				*	*							
			*				*	*								
Capretti et al. [12]		2014						*						*		
						*	*									
											*					
				*			*									
				*												
				*												
Miller [11]		2021									*					
											*					
				*												
Picone [22]		2013		*		*	*				*					
			*								*	CM, HVW				
						*	*					ACC, HK				
				*	*						*					
					*						*				*	
			*	*												
				*		*								*		
				*												
			*													
					*		*									
								*			*	PCE				
				*	*						*					
						*						Abnormal gyration				
					*			*								
					*	*	*							*	*	
			*	*				*	*							
					*	*										
							*									
			*						*		*					
			*				*		*		*					
Picone [23]		2008						*								
								*								
				*												
				*												
											*					
											*					
											*					
				*												
				*			*									
				*			*									
								*								
				*												
				*							*					
							*				*				*	
					*							HVW				
				*	*											
					*		*									
					*											
				*		*						PHV				
				*		*						PVH				
						*						PVH				
							*								*	
							*								*	
				*			*				*	PVH				
						*	*	*				PVH				
Leruez-Ville [24]		2021						*	*		*					
				*				*								
											*					
				*							*					
				*				*	*							
				*							*					
							*	*				LSV			*	
				*								LSV			*	
				*				*							*	
				*		*						PH				
											*					
											*					
											*					
											*					
											*					
											*					
							*				*					
								*								
											*					
											*					
											*					
											*					
											*					
Benoist [25]	2008				*	*										
							*									
						*										
					*		*									
							*					HVW				
							*									
							*					HVW				
							*									
					*	*										
					*		*									
					*	*										
						*										
							*									
							*									
Sanchez [26]	2016						*			*	*					
Mathias [27]	2019		*	*			*			*	*					
Bokström [28]	2010			*						*						
Henrich [29]	2002		*		*		*									
Ortiz [30]	2004		*			*	*				*					
Salmaso [31]	2006															
Nigro [32]	2002			*			*			*						
Lazzarotto [33]	2014			*			*	*			*					
Erenel [34]	2024		*	*					*		*					
											*					
					*					*	*	PCE				
											*					
							*									
			*	*					*	*						
								*								
							*									
							*	*								

Abbreviation: HB = hyperechogenic bowels, IUGR/SGR = intrauterine growth retardation/small for gestational age, PV cyst = periventricular cyst, SE cyst = subependymal cyst, CM = cardiomegaly, PH = polyhydramnios, HVW = hyperechogenic ventricular walls, ACC = agenesis of corpus callosum, HK = hyperechogenic kidney, PCE = pericardial effusion, PVE = periventricular hyperechogeny, LSV = lenticular striated vasculopathy, * could be observed.

**Table 2:** Ultrasound abnormalities summarized.

	n
Fetal hydrops	21
Hyperechogenic bowels	39
Intracranial calcification	19
Microcephaly	19
Hepatomegaly	27
Placentomegaly	11
Oligohydramnios	8
IUGR/SGR	47
Ventriculomegaly	51

**Table 3:** MRI abnormalities.

	n
Pseudocyst	4
Periventricular cyst	1
Subependymal cyst	20
Hypoplastic cerebellum	4

**Table 4:** Complications with hearing loss.

	n
Negative	112
Mild	42
Severe	19

**Table 5:** Association between prenatal ultrasound signals and postnatal hearing loss.

Hydrops	0.1426
Hyperechogenic bowels	0.1337
Ventriculomegaly	0.0052
Hepatosplenomegaly	1.0000
IUGR/SGR	0.8623

**Table 6:** Summary of neurological complications.

	n
Epilepsy	4
ADHD	4
Cerebral palsy	6
Motor delay	8
Cognitive delay	8
Verbal delay	15

**Table 7:** Association between prenatal ultrasound signals and postnatal neurological complications.

Ventriculomegalia	0.20127089
Subependymal cyst	0.00003547

## Data Availability

All data are available upon request.

## Abbreviations

The following abbreviations are used in this manuscript:

CMV	Cytomegalovirus
MRI	Magnetic resonance imaging
CT	Computer tomography
TOP	Termination of pregnancy
IUGR	Intrauterine growth restriction/retardation
SGR	Small for gestational age
